# Re-thinking Innovation in Organizations in the Industry 4.0 Scenario: New Challenges in a Primary Prevention Perspective

**DOI:** 10.3389/fpsyg.2018.00030

**Published:** 2018-01-31

**Authors:** Letizia Palazzeschi, Ornella Bucci, Annamaria Di Fabio

**Affiliations:** Department of Education and Psychology, Psychology Section, University of Florence, Florence, Italy

**Keywords:** innovation, technological innovation, psychological innovation, innovative work behavior, organizations, primary prevention perspective

## Abstract

In organizations, innovation is considered a relevant aspect of success and long-term survival. Organizations recognize that innovation contributes to creating competitive advantages in a more competitive, challenging and changing labor market. The present contribution addresses innovation in organizations in the scenario of Industry 4.0, including technological innovation and psychological innovation. Innovation is a core concept in this framework to face the challenge of globalized and fluid labor market in the 21st century. Reviewing the definition of innovation, the article focuses on innovative work behaviors and the relative measures. This perspective article also suggests new directions in a primary prevention perspective for future research and intervention relative to innovation and innovative work behaviors in the organizational context.

## Introduction

In organizations, innovation is considered a relevant aspect of success and long-term survival (Anderson et al., 2014). Organizations recognize that innovation contributes to creating competitive advantages (West, 2002a; Zhou and Shalley, 2003; Anderson et al., 2004; Lukes and Stephan, 2017). The process of innovation relates to generating and implementing new ideas, processes, and procedures to perform tasks in the best, most effective manner and offer the best products and services (Hammond et al., 2011; Lukes and Stephan, 2017). The innovation process includes technological innovation and psychological innovation (Anderson et al., 2004; Hammond et al., 2011).

Technological innovation comprises “new products and processes and significant technological changes of products and processes” (Organization for Economic Co-operation, and Development [OECD], 2001). Mentz (2006, p. 12) proposed a working definition of technological innovation that includes three aspects: invention, “to conceive and produce a new solution (from a scientific and technological knowledge) to a real or perceived need” (p. 12); realization, “to develop this solution into a viable and produceable entity”; and implementation, “to successfully introduce and supply this entity to the real or perceived need.”

Psychological innovation is focused on the characteristics of the innovator,innovative behaviors, and psychological mechanisms that guide innovation (Anderson et al., 2004; Kumar and Bharadwaj, 2016). In the literature, not only the implementation of technological systems as technological innovation emerges but also, and above all, the development of innovative behaviors (Scott and Bruce, 1994; Janssen, 2000; Felin et al., 2015; Lukes and Stephan, 2017) and of a culture of innovation (Patterson et al., 2005; Reicher, 2011) shared by workers with the aim of maintaining the introduced innovations. This is claiming the value of a psychological innovation with and beyond technological innovation (Baer and Frese, 2003; Bhatnagar, 2012).

## Technological and Psychological Innovation in Industry 4.0

In Industry 4.0, innovation is a core concept. Industry 4.0 refers to the trend of increased use of information and automation technologies in the manufacturing environment (Kagermann et al., 2013). Technological innovation is inherently implied in the scenario of Industry 4.0, a concept developed by the German Federal Government to enhance its high-tech strategy (Lasi et al., 2014). It is a multifaceted term that includes different interdisciplinary concepts (Lasi et al., 2014). In fact, in some cases, Industry 4.0 is used as a synonym for the Fourth Industrial Revolution, considering its technological potential (similar to that introduced by the first industrial revolution) in mechanization, use of electricity, and the beginning of digitalization (Lasi et al., 2014). From a technical perspective, Industry 4.0 is relative to increasing digitalization and automation of the manufacturing environment and the introduction of a digital value connection to increase communication between products and their environment and business partners (Brettel et al., 2014; Lasi et al., 2014; Schmidt et al., 2015).

Many advanced countries with economic systems based on the manufacturing industry must compete with emerging markets that have lower production costs (Lee et al., 2014). Manufacturing firms in advanced countries not only try to improve manufacturing technical innovation but also the modality of selling (Lee et al., 2014). They introduce a shift from simple product sales to an integration of products and services to deliver customer value (Baines et al., 2009; Lee et al., 2014). If technical innovation is essential for implementing Industry 4.0 in reality, then psychological innovation deserves more attention because it can make a difference (Bauer et al., 2015). It is no longer enough to focus on technical aspects; it is imperative to focus on employees (Bauer et al., 2015).

Manufacturing companies need new strategic approaches for holistic human resource management to cope with knowledge and competence challenges related to new technologies and processes of Industry 4.0 (Hecklau et al., 2016).

An analysis of the literature also shows that innovation can be facilitated by external social support with regard to the presence of more proximal supportive leaders and organizational support (House et al., 2004; Leung et al., 2005; Lukes and Stephan, 2017). Without these elements, innovation could be impeded. Leaders play an essential role in promoting innovation (Brisson-Banks, 2010). Research offers only some indications. On the one hand, some leadership styles (in particular, charismatic and transformational leaders) seem to inspire and motivate followers, promoting more innovation specifically at an ideation stage. On the other hand, different leadership styles (strategic leaders) seem to enhance organizational activity in general and decrease resistance to change, and therefore have a positive impact on implementing innovation and realizing effective transitions (Kesting et al., 2016). Organizational support includes the resources that organizations make available for implementing new ideas and encouraging innovations comprising top management support (Hunter et al., 2007; Lukes and Stephan, 2017). From the workers’ perspective, such organizational support for innovation encourages them to become involved in innovative behavior (Lukes and Stephan, 2017).

## The Complexity of the Construct of Innovation

The first definition of innovation in the workplace includes generating creative ideas at the first stage and implementing these ideas at the second stage (West and Farr, 1990). Subsequently, Scott and Bruce (1994) individuated three stages – idea generation, idea promotion, and idea implementation – as the development of adequate plans for the application of new ideas. Similarly, Janssen (2000) highlighted three stages, but his third stage is idea realization instead of idea implementation, underlining the passage from idea to its concrete realization, which is necessary for implementation. The three stages of innovation are thus: idea generation, idea promotion, and idea realization in terms of introducing innovative ideas into the work environment. More in-depth study individuates four stages of innovation: (1) idea generation, which means to develop novel and potentially useful ideas; (2) idea promotion, with the aim to sell an idea to others and to find supporters for an idea; (3) idea realization that is relative to the concretization of an idea into the work environment; (4) idea implementation, a successful introduction of the innovative idea into work contexts (Anderson et al., 2004; Mentz, 2006).

Deepening the construct of innovation, an important distinction regarding the difference between innovation and creativity emerges (Hülsheger et al., 2009; Potocnik and Anderson, 2016). Regarding the two different innovation stages, idea generation and idea implementation (West and Farr, 1990; Hülsheger et al., 2009; Potocnik and Anderson, 2016), creativity is seen as the first stage of the process (idea generation); creativity can thus be considered a sub-process of innovation (West, 2002a,b; Hülsheger et al., 2009; Anderson et al., 2014). This perspective means that creativity mainly is focused on generating new ideas, while innovation principally centers on implementing ideas (West, 2002a,b). Therefore, creativity is relative to an absolute novelty, and innovation concerns ideas in which the novelty consists of being adopted and adapted from other organizations but used in a specific organization for the first time (Anderson et al., 2004).

Continuing to deepen the construct of innovation, it is important to consider similar but distinct constructs in the change and innovation literature. For example, these concepts include proactive behaviors (Crant, 2000; Ohly and Fritz, 2010; Potocnik and Anderson, 2016), job crafting (Wrzesniewski and Dutton, 2001; Wrzesniewski et al., 2010; Potocnik and Anderson, 2016), voice (Van Dyne and LePine, 1998; Potocnik and Anderson, 2016), taking charge (Morrison and Phelps, 1999; Potocnik and Anderson, 2016), personal initiative (Fay and Frese, 2001; Binnewies et al., 2007; Potocnik and Anderson, 2016), and extra-role behaviors (Van Dyne et al., 1995; Potocnik and Anderson, 2016).

It is also important to distinguish different levels of analysis regarding innovation: individual, team, and organization (Ramos et al., 2016). Analysis at an individual level is mainly relative to the study of innovative work behaviors (Ramos et al., 2016). This article will focus more in-depth on this level in defining innovative work behaviors and issues relative to their measurement.

In terms of the team level of analysis and its role in facilitating or inhibiting innovation in the workplace, it is important to consider different aspects in terms of antecedents as team input variables and team process variables and in terms of moderating influences on antecedent-criterion relationships (Hülsheger et al., 2009). Team input variables correspond to team composition and structure such as job-relevant diversity, background diversity, task interdependence, goal interdependence, team size, and team longevity (Hülsheger et al., 2009). Team process variables are relative to vision, participative safety, support for innovation, task orientation, cohesion, internal communication, external communication, task conflict, and relationship conflict (Hülsheger et al., 2009).

A recent meta-analysis (Hülsheger et al., 2009) showed the following results: team process variables of support for innovation, vision, task orientation, and external communication presented the most robust relationships with innovation; team input variables showed weaker effect sizes. Regarding moderators, analyses showed that relationships are different based on measurement method (self-ratings vs. independent ratings of innovation) and measurement level (individual vs. team innovation). Team variables displayed considerably stronger relationships with self-report measures of innovation compared with independent ratings and objective criteria. Team process variables were more associated with innovation at the team level rather than the individual level. These results suggest the importance to be focused on offering to the group high support for innovation and creating a climate opened to change in an intervention perspective (Hülsheger et al., 2009).

According to an organizational level, innovation is positively associated with management-related factors such as the following: management support and cooperative conflict management (Jung et al., 2003, 2008; Elenkov and Manev, 2005; Damanpour and Schneider, 2006; Choi and Chang, 2009); knowledge search and spillover (transfer), knowledge stock, social network (Katila and Ahuja, 2002; Kyriakopoulos and De Ruyter, 2004; Belenzon and Berkovitz, 2010; Kijkuit and van den Ende, 2010; Operti and Carnabuci, 2014); organizational structure as harmonization, decentralization, reorganization (Damanpour and Schneider, 2006; Shipton et al., 2006; Cohendet and Simon, 2007; Vermeulen et al., 2007; Jung et al., 2008; Karim, 2009); organization strategy as innovation strategy (He and Wong, 2004; Richard et al., 2004; Un and Cuervo-Cazurra, 2004).

## Focusing on Innovative Work Behavior

Alongside these attempts to define innovation, the need to focus on translating innovation in work behaviors of employees emerged (Ramos et al., 2016). According to West and Farr (1990, p. 9), innovative work behavior refers to “the intentional introduction and application within a role, group or organization of ideas, processes, products or procedures, new to the relevant unit of adoption, designed to significantly benefit the individual, the group, the organization or wider society.” Subsequently, Scott and Bruce (1994) described innovative work behavior as generating creative ideas, promoting ideas to others, and developing adequate plans to implement new ideas.

In 2000, Janssen underlined three aspects of innovative work behavior: idea generation, idea promotion and idea realization. Until then, the construct of innovative work behavior was considered essentially unidimensional as relative measures. In detail, the innovative work behavior measure (Scott and Bruce, 1994) is composed of six items (e.g., generate creative ideas, promote ideas to others, develop plans for implementing new ideas), with a Cronbach’s alpha of 0.89. The innovative work behavior scale (Janssen, 2000) is composed of nine items on the three basic steps in the innovation process: idea generation (creating new ideas for difficult issues), idea promotion (acquiring approval for innovative ideas), idea realization (introducing innovative ideas into the work environment in a systematic way). These three components are considered part of an overall scale of innovative work behavior due to their high intercorrelations, with a Cronbach’s alpha of 0.95 for self-ratings and 0.96 for the supervisor ratings.

Subsequently, Kleysen and Street (2001) affirm that unidimensional measures do not sufficiently capture the richness of the construct, and introduced a multidimensional structure with five dimensions: (1) opportunity exploration (three items, example: “Look for opportunities to improve an existing process, technology, product, service or work relationship”); (2) generativity (two items, example: “Generate ideas or solutions to address problems”); (3) formative investigation (three items, example: “Experiment with new ideas and solutions”); (4) championing (three items, example: “try to persuade others of the importance of a new idea or solution”); (5) application (three items, example: “Implement changes that seem to be beneficial”). Because the theoretical structure was not confirmed through the empirical analysis, Kleysen and Street (2001) presented a unidimensional scale composed of 14 items with a Cronbach’s alpha of 0.95.

The multidimensionality of the innovative work behavior construct emerged empirically in other scales. The Krause (2004) measure individuates two dimensions: generation and testing ideas (five items, Cronbach’s alpha 0.78) and implementation (three items, Cronbach’s alpha 0.82). Exploratory factor analysis shows two factors as factorially distinct. The De Jong and Den Hartog (2010) measure detects four dimensions: opportunity exploration (two items, example: “Pay attention to issues that are not part of his daily work,” Cronbach’s alpha 0.88); idea generation (three items, example: “Generate original solutions for problems,” Cronbach’s alpha 0.90), idea championing (two items, example: “Attempt to convince people to support an innovative idea,” Cronbach’s alpha 0.95), and idea implementation (three items, example: “Contribute to the implementation of new ideas,” Cronbach’s alpha 0.93). However, the factorial structure is weak with two dimensions with only two items.

To overcome the limitations of the existing measures, Lukes and Stephan (2017) developed the Innovative Behavior Inventory, a multidimensional measure composed of seven dimensions with a good factor structure to evaluate the different aspects of the construct: (1) Idea generation (three items, example: “I try new ways to do things at work,” Cronbach’s alpha 0.67); (2) Idea search (three items, example: “I try to get new ideas from colleagues or business partners,” Cronbach’s alpha 0.81); (3) Idea communication (four items, example: “When I have a new idea, I try to persuade my colleagues of it,” Cronbach’s alpha 0.72); (4) Implementation starting activities (three items, example: “I develop suitable plans and schedule for the implementation of new ideas,” Cronbach’s alpha 0.61); (5) Involving others (three items, example: “When I have a new idea, I look for people who are able to push it through,” Cronbach’s alpha 0.70); (6) Overcoming obstacles (four items, example: “I’m able to persistently overcome obstacles when implementing an idea,” Cronbach’s alpha 0.88); (7) Innovation outputs (three items, example: “Many things I come up with are used in my organization,” Cronbach’s alpha 0.78). The multidimensional structure was confirmed through confirmatory factor analysis. From the analysis of the literature emerges the necessity to continue to study the dimensionality of the construct, and perhaps include more aspects regarding leader and organizational support.

## Conclusion

Innovation is a key driver that can guarantee competitive advantages for organizations (Lukes and Stephan, 2017), but it is crucial to identify and consider not only technological innovation but also psychological innovation. In particular, it is necessary not only to implement technological systems as technological innovation but above all to develop innovative work behaviors (Scott and Bruce, 1994; Janssen, 2000; Felin et al., 2015; Lukes and Stephan, 2017). In this regard, it seems that so far, there is not a primary prevention perspective focused on the early enhancement of individual strengths balanced with risk reduction (Hage et al., 2007; Kenny and Hage, 2009; Di Fabio and Kenny, 2016b). At the individual level, this perspective mainly calls for workers preventively equipped with resources (Di Fabio and Palazzeschi, 2012; Di Fabio, 2014; Di Fabio et al., 2017) to be developed with specific early training. At the organizational level, it calls for constructing and facilitating an organizational climate that supports change and developing leaders equipped with specific skills for favoring change and accepting it adaptively. This perspective also includes a need for managing workers with new abilities to increase flexibility, resilience, and enthusiasm for the novelties and engaging themselves in something often not known.

By presenting some current new instruments and training in this perspective, it is possible, at an individual level, to introduce preventive and new variables in relation to innovative work behaviors, such as acceptance of change (Di Fabio and Gori, 2016a) or workplace relational civility (Di Fabio and Gori, 2016b). Until now, the focus was only on resistance to change; in a positive primary preventive perspective, acceptance of change has been introduced as predisposition to change, support for change, change seeking, positive reaction to change, and cognitive flexibility (Di Fabio and Gori, 2016a). Another variable that could be worthy of interest in this perspective, since until now the focus was on workplace incivility, is workplace relational civility that includes relational decency, culture, and readiness (Di Fabio and Gori, 2016b). Relational civility could contribute to increased innovative behaviors in organizations through introducing a new form of organizational relationality for innovation and sustainability (Di Fabio and Kenny, 2016a; Di Fabio et al., 2016; Di Fabio, 2017a,b). Thus, workplace relational civility also brings the focus to the organizational level because it can be considered a basis to create a work climate open to change, building mutual trust and focusing on offering high support for innovation (Hülsheger et al., 2009). Furthermore, at an organizational level, it could be interesting to reflect on leadership styles needed to promote innovative work behaviors, a culture for change, and for adaptively building the unknown future chapter of each organization. More traditionally, research underlines the role of transformational leadership in promoting innovative work behaviors (Kesting et al., 2016), inspiring and motivating followers. It could be interesting to study new forms of leadership in relation to innovative work behaviors and their effectiveness/efficiency in different contexts of organizational support. These forms of leadership, for the moment, are referred to servant leadership (Ehrhart, 2004), benevolent leadership (Cheng et al., 2004; Wang and Cheng, 2010), authentic leadership (Avolio et al., 2009), ethical leadership (Gallagher and Tschudin, 2010), and mindful leadership (George, 2012; Herold, 2013). Based on previous reflections, there is also a need for innovative leadership styles. Also, future perspectives regarding innovative work behaviors in a primary prevention framework call for new interventions and specific training validated through the use of control groups to promote individual strengths for sustaining innovation and innovative work behaviors.

## Author Contributions

LP and AD conceptualized the work and ideated the structure. LP, OB, and AD analyzed the literature, and all authors wrote the manuscript. Then all authors read and revised the manuscript several times.

## Conflict of Interest Statement

The authors declare that the research was conducted in the absence of any commercial or financial relationships that could be construed as a potential conflict of interest.
